# An Approach to Enhance Dissolution Rate of Tamoxifen Citrate

**DOI:** 10.1155/2019/2161348

**Published:** 2019-01-20

**Authors:** Nagaraja SreeHarsha, Jagadeesh G. Hiremath, Swathi Chilukuri, Rajesh Kumar Aitha, Bandar E. Al-Dhubiab, Katharigatta N. Venugopala, Abdullah Mossa Alzahrani, Girish Meravanige

**Affiliations:** ^1^Department of Pharmaceutical Sciences, College of Clinical Pharmacy, King Faisal University, Al-Ahsa, Saudi Arabia; ^2^Department of Pharmaceutics, Oxbridge College of Pharmacy, Herohalli Cross, Magadi Road, Bangalore, India; ^3^Department of Pharmaceutics, East West College of Pharmacy, Bangalore-560 091, Karnataka, India; ^4^Hetero Drugs Ltd, 7-2-A2, Hetero Corporate, Industrial Estates, Sanath Nagar, Hyderabad-500 018. Telangana, India; ^5^Department of Biotechnology and Food Technology, Durban University of Technology, Durban 4001, South Africa; ^6^Department of Biological Sciences, College of Science, King Faisal University, Al-Ahsa, Saudi Arabia; ^7^Department of Biomedical Sciences, College of Medicine, King Faisal University, Al-Ahsa, Saudi Arabia

## Abstract

We tested the solubility and dissolution of tamoxifen citrate to ascertain the optimal conditions for faster dissolution. Using the solvent evaporation method and hydrophilic carriers, we formulated tamoxifen citrate (TC) that contained solid dispersions (SDs). We increased the solubility and dissolution rate of TC with a solid dispersion system that consisted of polyethylene glycol (PEG-6000), beta-cyclodextrin (*β*-CD), and a combination of carriers. Physicochemical characteristics of solubility (mg/ml) were found to be 0.987±0.04 (water), 1.324±0.05 (6.8pH PBS), and 1.156±0.03 (7.4 pH PBS) for F5 formulation, percentage yield was between 98.74 ± 1.11% and 99.06 ± 0.58%, drug content was between 98.06±0.58 and 99.06±1.10, and dissolution studies binary complex showed a faster release of TC as compared to a single polymer and pure drug. Furthermore, thermal properties, physicochemical drug and polymer interaction, crystal properties, and morphology were determined using differential scanning calorimetry (DSC), infrared spectroscopy (FT-IR), X-ray differential studies, and scanning electron microscopy. We used the same proportion of carrier concentrations of the formulations to calculate the solubility of TC. Our results demonstrated that increased concentrations of *β*-C yielded an improved solubility of TC, which was two times higher than pure TC. The uniformity in drug content was 97.99 %. A quicker drug release occurred from the binary complex formulation as seen in the dissolution profile. FTIR demonstrated an absence in the physicochemical interaction between the drug and carriers. The drug was also found to be dispersed in the amorphous state as revealed by DSC and XRD. The drug concentration did not vary during various storage conditions. Our* in vivo* studies demonstrated that SD displayed significantly higher values of C_max_ (p < 0.05) and AUC_0-24_ (p < 0.05) as compared to free TC. Furthermore, T_max_ in SD was significantly lower (p < 0.05), as compared to free TC.

## 1. Introduction

Pharmaceutical drugs with poor solubility, bioavailability, permeability, and rapid metabolism and elimination comprise a large percentage of drugs in the pharmaceutical market today. During the preformulation stage of drug development, the physicochemical properties of pharmaceutical drugs present a continuing challenge. As such, large efforts have been mounted in the search for optimal techniques that can yield drugs with improved solubility and dissolution and, consequently, better drug efficacy. Solid dispersion is one such technique that is currently under investigation for use in the improvement of the solubility of active pharmaceutical ingredients (API). It is currently estimated that 40–60% of pharmaceutical compounds are more lipophilic as opposed to hydrophilic [1, 2]. Tamoxifen citrate (TC) is a perfect example of a poorly soluble compound. TC is currently indicated as an adjuvant endocrine therapy used in the treatment of hormone-sensitive and early-stage breast cancer. The poor solubility of TC can be seen from its physicochemical characteristics; solubility in water is 0.3 mg/L at 20°C (pH 3.0–3.5) and in 0.02 N HCl is 0.2 mg/ml at 37°C. Furthermore, it is indicated to be soluble in ethanol and methanol and is slightly soluble in acetone and chloroform (Sigma Product Information for product T9262, available at http://www.sigma.com). The typical dose for tamoxifen treatment is between 20 and 40 mg daily for 5 years [3]. Unfortunately, this prolonged period of treatment and accumulation does present an increased probability of hepatotoxicity and endometrial cancer due to its long half-life of 5–7 days [3–5]. Furthermore, the bioavailability of TC has been previously reported to be in the range of 20–30% [6]. Previous efforts to increase the solubility of TC have included the use of 0.5% w/v sodium lauryl sulfate alongside phosphate-buffered saline (PBS, pH 7.4) [7]. Furthermore, additional studies have demonstrated the use of hydroxyl propyl-*β*-cyclodextrin to improve the solubility of tamoxifen base, as well as TC, hydroxyl propyl-*β*-cyclodextrin [8, 9]. Here, we present a study on an optimization protocol aimed at improving the solubility and dissolution of TC.

To increase the solubility and/or dissolution rate, we first converted the drug into its amorphous form using solid dispersions and hydrophilic carriers [10]. From the various techniques present in the field, solid dispersion is perhaps one of the best available which can increase the solubility and dissolution of drugs. However, previous work has also demonstrated that the solvent evaporation method, which increases mixing at the molecular level, allows for the matrix material and drug to act as a dissolved solvent system. Using this method, the solubility of lipophilic drugs has been greatly increased and is currently used industrially [11]. Yet, many researchers prefer the use of hydrophilic carriers to deliver lipophilic drugs. Some of these carriers include *β*-cyclodextrin (*β*-CD) and polyethylene glycol (PEG-6000), and these have been used to increase the solubility of a variety of drugs [10, 12–16].

Solid dispersions (SD) can be prepared using polyethylene glycols (PEGs), which have a molecular weight of 1,500–20,000. While the solubility of PEGs is typically good in water, it does decrease with an increase in its molecular weight. Furthermore, it has the advantage of increased solubility in a wide range of organic solvents. Each of the PEGs of interest which include PEG-1000, PEG-4000, PEG-6000, and PEG-20,000 has a melting point of under 65°C, and each has a range of 30–40, 50–58, 55–63, and 60–63°C, respectively. Furthermore, their low melting point makes it feasible to use a melting method when manufacturing SDs. They also improve wettability. Interestingly, among the PEGs, the SDs of drugs with PEG-6000 lack many of the problems of stability, solubility, dissolution, and bioavailability. They also demonstrate increased safety.

SDs can also be prepared using cyclodextrins (CDs), which are cyclic oligosaccharides that consist of 6–8 glucose units linked by the 1,4-*α*-glucosidic bonds. This special structure is due to the fact that it has 7 glucose units and a highly active hydroxyl group located on its surface, which allows for easy chemical modification.

Till date, there are relatively few established methods for the use of both PEG-6000 and *β*-CD combinations that contain TC-dispersed solids. Here, we prepared TC-dispersed solid dispersion products using the solvent evaporation method. We measured various in vitro characteristics such as solubility, physicochemical properties, and dissolutions. Furthermore, pharmacokinetics studies were executed for evaluation in animal model.

## 2. Materials and Methods

We obtained TC as a gift from Khandelwal Laboratories Mumbai, India. All analytical-grade chemicals and solvents, including *β*-cyclodextrin (*β*-CD) and polyethylene glycol (PEG-6000), were purchased from Sigma-Aldrich (Bangalore, India).

### 2.1. Solubility Studies

We performed a solubility test, in which we added an excess of TC, ~50mg, in 25ml of water and phosphate-buffered saline (pH 6.8, 7.4 PBS), with and without the inclusion of carriers. Physical mixtures (PMs) were prepared using a mortar with the same proportion of the concentration of carrier formulations as outlined in Table 1. All solutions were vortexed for 5 mins and placed in a bench top water bath shaker (Remi, India) at an angular acceleration of 100 rpm for 24 h at 37±1°C. After an incubation of 24 hrs, the samples were vortexed for 5 min and centrifuged at 10,000 rpm at 37±1°C. The supernatant sample was obtained and diluted with the different solvent systems, and samples were analyzed using high-performance liquid chromatography (HPLC). The HPLC system was comprised of a Shimadzu SPD-10ATVP, a binary pump with a sample injector SPD-10AVP, a variable UV wavelength detector, and a Spincotech station for data analysis. 50 *μ*L was injected into Phenomenex C-8 column, (4.6×250 mm, 5 *μ*m) and Phenomenex C-8 guard column cartridge (KJ0-4282, 4.0×3.0 mm, 5 *μ*m), and methanol was used as the mobile phase (PBS, pH 7.4 (9:1)) with a flow rate of 1 mL.min-1. The effluent was detected at a wavelength of 277 nm. The efficacy of recovery was calculated by dissolving a known quantity of TC in methanol (PBS pH 7.4 (9:1)) and the above procedure was repeated [17]. Each combination was prepared in triplicate to determine the solubility of TC.

### 2.2. Preparation of Solid Dispersions

The solvent evaporation method was used to prepare TC-dispersed solid dispersions. Preparations of TC with either PEG, *β*-CD, or the combination of the two were dissolved in methanol and acetone (3:1). This was followed by evaporating the solvents at room temperature with continued stirring at 1,000 rpm. Afterwards, dispersions were stored at a temperature of 40°C for 24 hrs in an oven to ensure complete evaporation of solvents. Dispersions were then pulverized with mortar and pestle, and physicochemical characteristics were extracted and analyzed [18] (Table 2).

### 2.3. Drug Content

Equivalent amounts of 10 mg drug containing TC solid dispersions were dissolved in a known amount of methanol and sonicated for 10 mins. Using a 0.22 *μ* membrane filter, all solutions were filtered. The filtrate was diluted with different aqueous media systems, water and PBS (pH 6.8 and 7.4). The drug content was determined by HPLC method [17]. Triplicate results were reported.

### 2.4. In Vitro Dissolution Studies

We used the United States of Pharmacopeia (USP) rotating paddle method to determine the release profile of the solid dispersions by using different systems of water and PBS (pH 6.8 and 7.4), respectively. Formulations equivalent to 20 mg of TC were added to 500 ml of dissolution media at 37±0.5°C at a speed of 100 rpm. 10 ml of the sample was withdrawn from the aliquot at predetermined time intervals. An equivalent amount of fresh medium was also replaced to maintain the conditions of the sink. Furthermore, withdrawn samples were prepared with suitable dilutions (as described above in solubility section) to determine the content of the TC [19].

### 2.5. Differential Scanning Calorimetry (DSC)

2–4 mg of TC, PEG-6000, *β*-CD, TC+PEG, TC+*β*-CD, TC+PEG-6000+*β*-CD, and their formulations were placed in an aluminum pan, sealed with an aluminum cap, and stored using the nitrogen purging method. Samples were scanned at 10–300°C at a rate of 10°C/min using differential scanning calorimeter [20] (Mettler-Toledo DSC, USA).

### 2.6. X-Ray Diffractometer (XRD)

An X-ray diffractometer (Philips, UK) was used to record the powder XRD of TC, PEG-6000, *β*-CD, TC+PEG, TC+*β*-CD, TC+PEG-6000+*β*-CD, and solid dispersions. The scanning rate used was 5/min, and the diffraction angle (2*θ*) was 0–70 [21].

### 2.7. Fourier Transforms Infrared Spectroscopy (FT-IR)

All samples were prepared in the form of KBr pellets and scanned from 4000 to 400 cm-1 using Fourier Transform Infrared (FT-IR) spectrophotometer [22] (Thermo Nicolet Avatar 370, Japan).

### 2.8. Scanning Electron Microscopy (SEM)

Surface morphological studies were carried out using SEM on the selected formulations (F1, F2, F4, and F5) (JSM-848, Joel, Japan). Samples were dried, glued to aluminum sample holders, and then gold-coated using argon. The surface morphology of the coated samples was then analyzed under magnification of 1000x to 10,000x with set voltage of 20 kV [23].

### 2.9. Stability Studies

Stability studies of SD (F5) were performed in a stability chamber according to ICH guidelines 40°C/75% RH for a 6-month period. The drug contents at 0, 3, and 6 months were analyzed to see the effect of storage conditions. Furthermore, samples were prepared with suitable dilutions (as described above in solubility section) to determine the content of the TC [19].

### 2.10. In Vivo Pharmacokinetics Studies

Female Sprague-Dawley rats averaging an average of 250 ± 20 gm and age range of 4-5 weeks were purchased from the local market in Al-Ahsa, Saudi Arabia. All rats were maintained in a light controlled room with a temperature of 20°C ± 2° and relative humidity of 55% RH ± 5% RH. All rats were divided into 2 groups (n = 6) after an overnight fast (12 h) with free access to water before the experiments. The first group of animals received an oral suspension of TC, while the second received oral TC with optimized solid dispersion (1:2:6, TC: PEG-6000: *β*-CD). Both groups received TC at a dose of 10 mg/kg body weight. After one hour after administration, rats were anesthetized using diethyl ether and blood samples collected via a retroorbital puncture in regular intervals of 1, 2, 3, 4, 5, 6, 8, 12, and 24 hrs after dosing.

To separate plasma from red blood corpuscles (RBCs), withdrawn blood samples were placed in heparinized Eppendorf tubes and centrifuged at 2000 rpm for 5 mins [24]. The samples of plasma were then stored at -80°C till analysis time. HPLC was used to analyze the plasma levels of TC in which the mobile phase was acetonitrile: methanol (85:15% v/v) which contained 0.02% triethylamine and enacted at a flow rate of 1.5 ml/ min [21]. To extract the drug from plasma, acetonitrile was added to each sample (1:4 volume) followed by a 30-second vortex and centrifugation at 4,000 rpm for 15 minutes. The upper layer of the centrifuged sample was withdrawn, filtered through 0.45 *μ*m Millipore filter. 10 *μ*l of the filtrate was then injected into the HPLC column (Phenomenex C-8 column (4.6×250 mm, 5 *μ*m) and Phenomenex C-8 guard column cartridge (KJ0-4282, 4.0×3.0 mm, 5 *μ*m)). Blank plasma was infused with 0.1 ml of TC standard solution and treated in the same way as the test samples to develop the standard curve. The measurement was done with a UV detector (Shimadzu SPD-10ATVP, Japan) at 277 nm. These studies were conducted according to the University Animal Ethical Committee protocol, College of Clinical Pharmacy, King Faisal University, Saudi Arabia (approval number: VET/KFU/28/PR-3145).

### 2.11. Statistical Analysis

All experiment measurements were triplicated and values are represented as the mean ± SD unless otherwise stated.

## 3. Results and Discussion

The goal of our study was to garner a set of optimal parameters which would increase the solubility and dissolution properties that yield reproducible solid dispersions of TC. We determined the solubility of TC using a previously developed method [17] and validated the previously reported linear correlation coefficient (R2) of 0.9999, eluted at a retention time of 7.02 ± 0.3 min, which was with respect to intraday and interday precision and accuracy. Our results demonstrated a percentage coefficient of variation (CV) of 2.6% and 3.1% at the lowest drug concentration, when compared to the highest drug concentration of 0.94% and 0.79%. With an increase in the concentration of polymers, all the prepared samples demonstrated a substantial increase in their solubility (Table 1). Furthermore, the formulations of *β*-CD demonstrated a higher solubility profile compared to PEG-6000. We attributed this finding to the improvement in the wetting effect, which in turn influences solid drug solution formation [10].

We used the solvent evaporation method to produce SDs. Using this method, we found a uniform distribution of the drug, which corresponded to a 98-99% recovery rate of the amount that was added to the formulations. Across all experiments, the percentage of drug recovery ranged from 97.40 ± 1.20% to 99.20 ± 0.42% (Table 2). As a result, these factors affected the degree of TC uniformity in all the solid dispersions. Furthermore, the evaporation of the solvent in the dispersion systems prevented the loss of drugs [11, 16].

**Figure 1:**
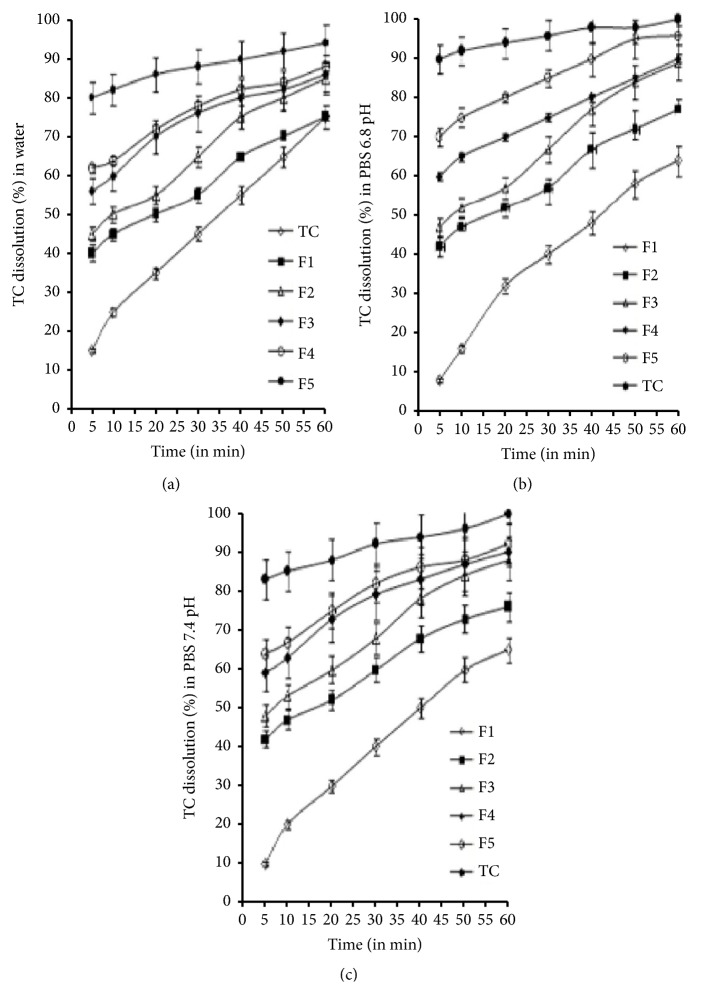
(a) Dissolution profiles of TC-PEG-6000 complexes carried out in water (◊) TC, (■) F1, (Δ) F2, (♦) F3, (*ο*) F4, and (●) F5. (b) shows the dissolution profiles of 6.8 pH PBS (◊) F1, (■) F2, (▲) F3, (*ο*) F4, and (●) F5. (c) shows the dissolution profiles of TC-PEG+*β*CD complexes carried out in 7.4, pH PBS (◊) F1, (■) F2, (▲) F3, (*ο*) F4, and (●) F5.

In Figures 1(a)–1(c), we show the dissolution profiles of TC-dispersed SDs. We compared similar factors by comparing the calculated dissolution efficacy in percentage (DE%) at 10, 20, and 30 min of TC release (Table 3). We found a significant difference during TC release between the individual polymers with binary complex formulations and pure TC. Interestingly, the formulations of the binary complex showed a faster release of TC as compared to a single polymer and pure drug. Similar results have been demonstrated by studies using TC inclusion complexes [8]. In our set of experiments, we interpret the enhanced dissolution rate as being due to the dispersion of TC at the molecular level [2, 13, 15]. Previous results have shown that the formation of solid drug solutions promotes immediate wettability and, as a result, better solubility [10]. We found this to also be the case in our results, which we interpret as being due to an increase in the surface area of the drug, which led to changes in the polymorphic results. Our interpretation was also supported by our results from DSC and XRD.

**Figure 2:**
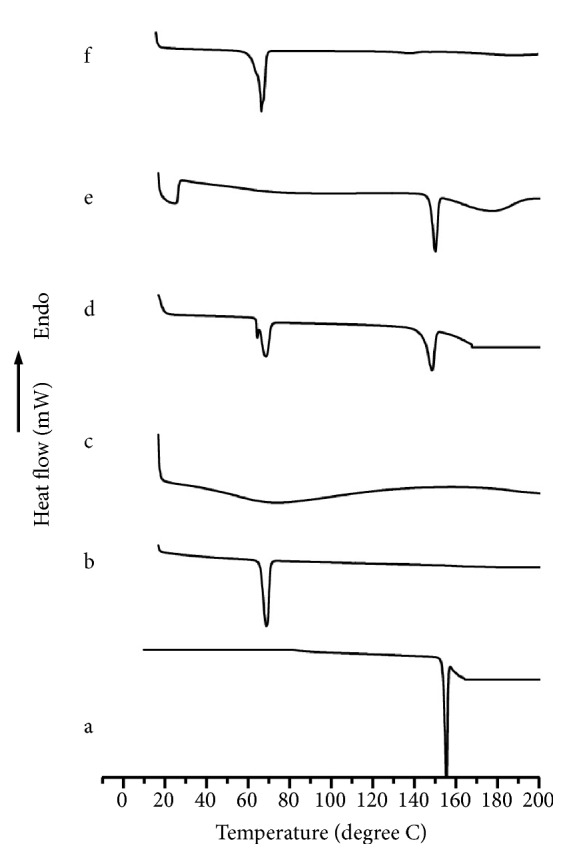
DSC thermograms of (a) PEG-6000, (b) *β*-CD, (C) TC+PEG-6000, (d) TC+*β*-CD, (e) TC+*β*-CD, and (f) TC+PEG-6000+*β*-CD (TC).

**Figure 3:**
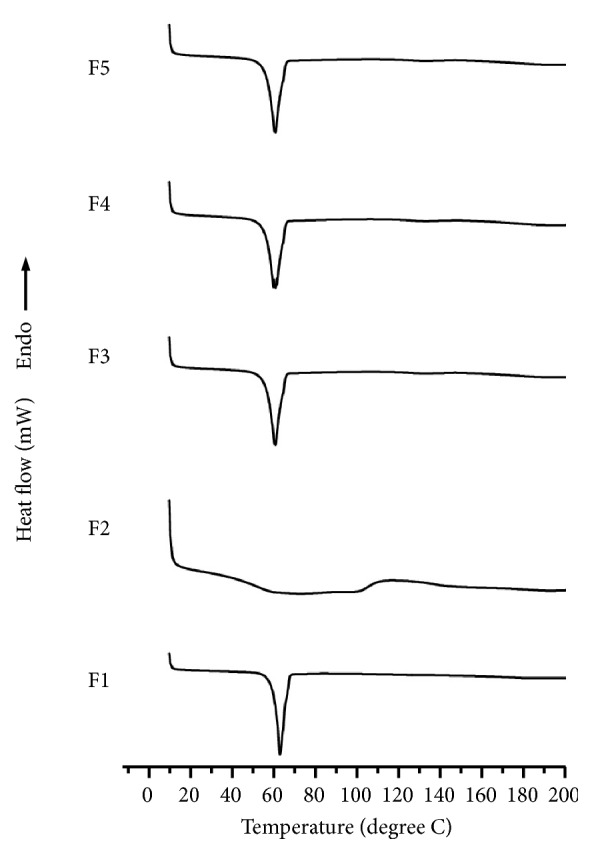
DSC thermograms of F1, F2, F3, F4, and F5.

There is a widespread use of DSC in the pharmaceutical industry to investigate the rapid and qualitative properties of solid dispersions. We used DSC to examine the temperature change of the TC formulations to assess the degree of drug solubilization. In Figure 2, we demonstrate the sharp endothermic peak of pure TC, which was reported to be at 148.61°C and corresponded to its melting point [17, 25]. On the other hand, the melting point of PEG-6000 was reported to be 63.24°C, while *β*-CD generated an endothermic peak at 68.21°C. We also examined the thermograms of the physical mixtures of TC+PEG and found an endothermic peak that appeared at 146.11°C and 63.54°C, while the TC+*β*-CD endothermic peak was detected at 147.51°C and 64.20°C. While there was a negligible change in temperature with the case of both physical mixtures, the physical mixture thermogram of TC+PEG-6000+*β*-CD clearly showed that the polymeric peak was only generated at 60.71°C, indicating the effect of high proportions of *β*-CD or drug solubilization in the molten polymer. We observed a similar pattern of thermograms in the solid dispersion of binary formulations and observed that the TC peak disappeared (Figure 3). We interpret that this phenomenon can be explained by the solubilization of the drug in the molten polymer that was dispersed in an amorphous nature as demonstrated in previous studies with similar carriers [26].

**Figure 4:**
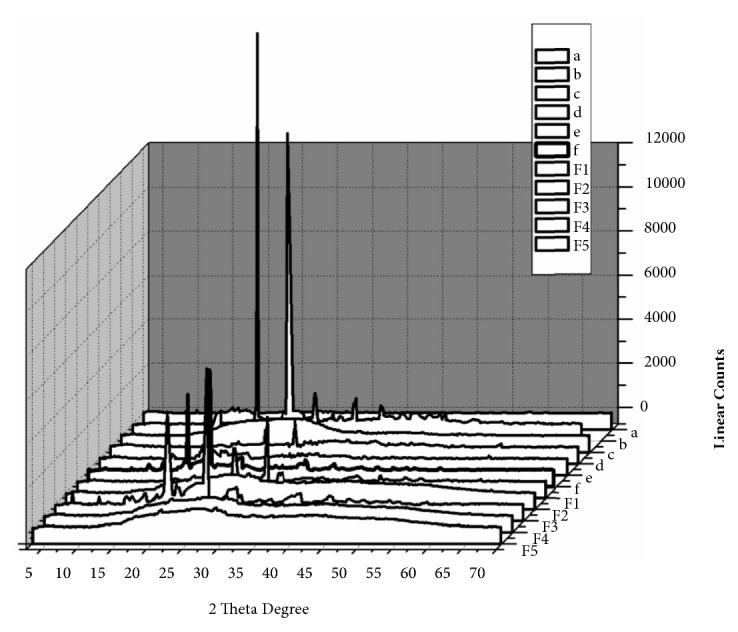
X-ray diffractograms of (a) TC, (b) PEG-6000, (c) *β*-CD, (d) TC+PEG-6000, (e) TC+*β*-CD, (f) TC+PEG-6000+*β*-CD, and F1, F2, F3, F4, and F5.

In Figure 4, we demonstrate that the pure TC exhibits numerous distinct diffraction peaks of high intensity of 2*θ* values of 9.24°, 13.56°, 15.98°, 17.49°, 18.77°, 20.16°, 21.06°, 24.06°, and 26.87°, which demonstrated that the drug is present in a highly crystalline state [17, 25]. PEG-6000 demonstrated high-intensity diffractions peaks of 2*θ* at 14.98°, 19.02°, 23.16°, 26.05°, and 30.76°, but, on the other hand, *β*-CD displayed peaks at 26.95° and 40.78°. These were all indicators of crystalline domains within the amorphous polymeric material [26]. The diffractogram of the PMs demonstrated all the characteristic peaks of TC; however, this was at a decreased level due to the low proportion of the drug in the carrier. Furthermore, the diffractograms of the SDs which were prepared by the solvent evaporation method demonstrated a different behavior when compared to PMs. This was indicated by the disappearance in the TC peaks, which are characteristics of a transition from a crystalline to an amorphous state. Similar characteristics have been reported for other drugs in the literature [26].

**Figure 5:**
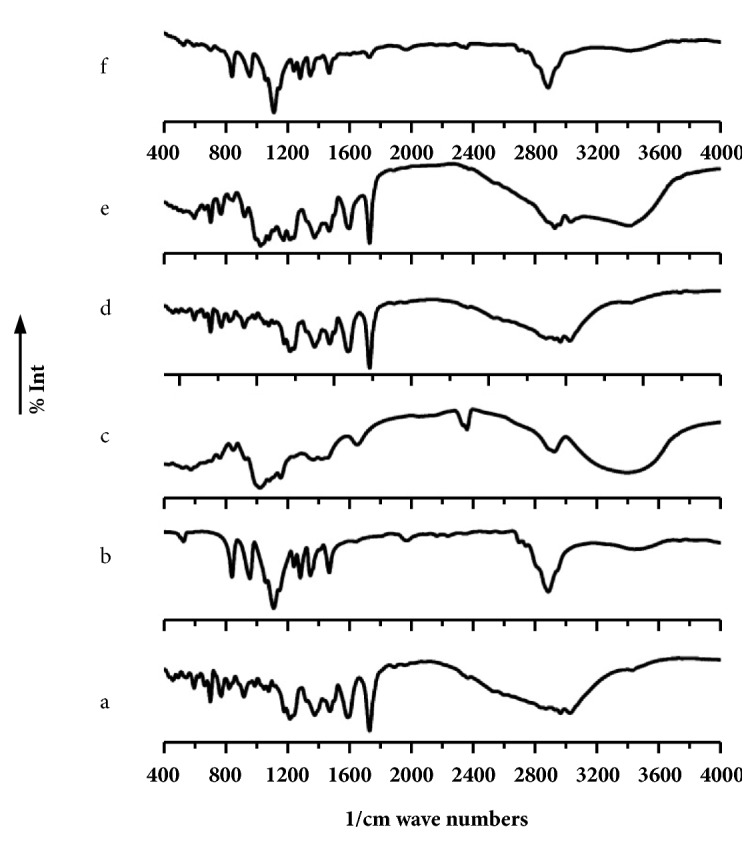
FTIR spectra of (a) TC, (b) PEG-6000, (c) *β*-CD, (d) TC+PEG-6000, (e) TC+*β*-CD, and (f) TC+PEG-6000+*β*-CD.

**Figure 6:**
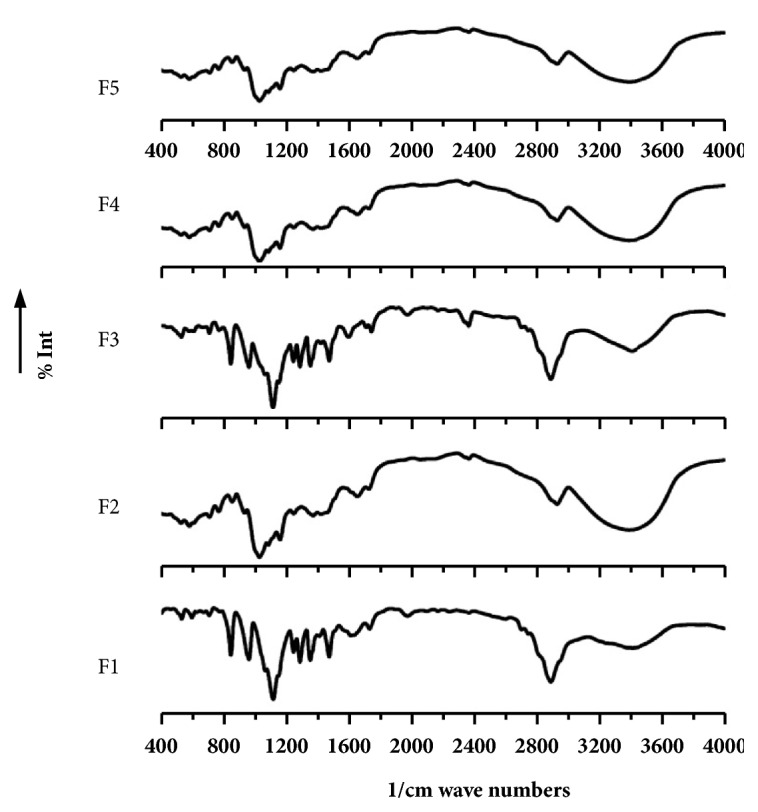
FTIR spectra of F1, F2, F3, F4, and F5.

To detect the possible interaction between the polymer ((PEG-6000), (*β*-cyclodextrin)) and TC or formulations, data from FT-IR were analyzed. In Figure 5, the FT-IR spectrum of the pure crystal form of TC demonstrated strong characteristic peaks at 3028–2872 cm-1 (C-H sp3 stretching), 1486 cm-1 (C=C ring stretching), and 1615 cm-1 (-NH bending) (Hiremath et al., 2012). The PEG exhibited characteristic peaks, 1510–1438 cm-1 (Ar-CH=O anhydride), 1420–1330 cm-1 (C-O-H bending), and 1159–1069 cm-1 (C-O and C-N ether and amine stretching); however, *β*-CD showed peaks at 2879 cm-1 (C-H stretching) and 1080 cm−1 (C-O bending), respectively. The absence of interactions between the drug and polymers was seen from the similar characteristics of drug peaks in the PMs, which also indicated the absence of interaction between the drug and polymers. Additionally, equal proportions of carrier formulations showed similar characteristics of drug peaks, which were maintained during the solid dispersion formation. On the other hand, the peaks were both broadened and of low intensity as compared to that of the PM. However, high proportions of *β*-CD (Figure 6) demonstrated decreased intensities due to the low concentration of the drug. This low concentration may have been due to either the analysis or the presence of the drug in the amorphous state or because the solvent method was used to prepare the drugs.

**Figure 7:**
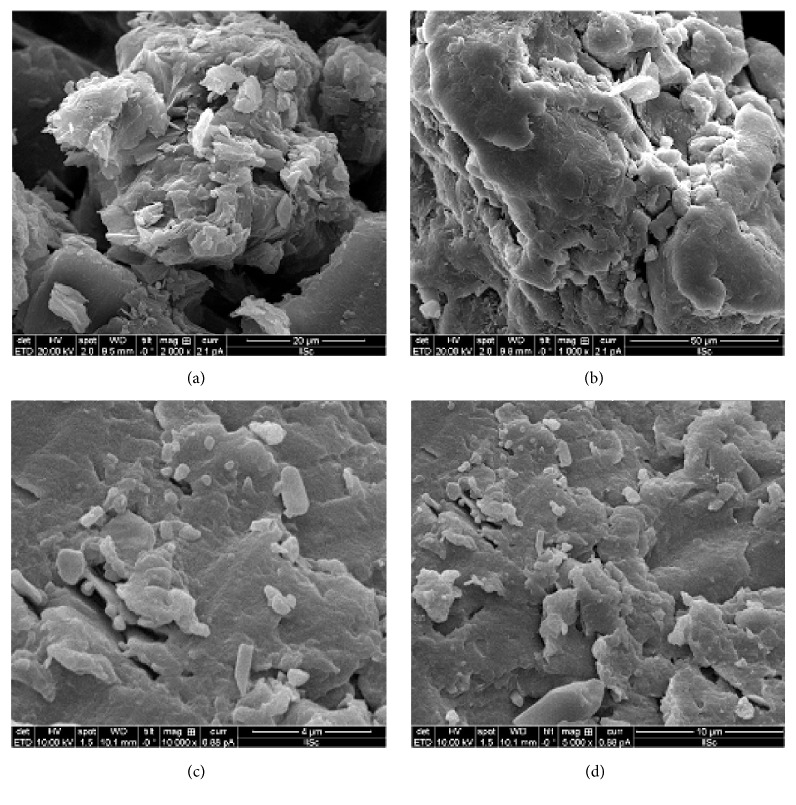
SEM microphotographs of solid dispersion of (a) F1, (b) F2, (c) F4, and (d) F5, respectively.

We carried out a SEM analysis to study the morphological structures of the formulated SDs. Obtained microphotographs of the solid dispersion formulations, F1, F2, F4, and F5, are shown in Figure 7. F1, which is a PEG-based formulation, showed a slightly dense aggregation of dense crystal particles, which affected the nonporous surface and possibly the solubility and dissolution of TC from solid-state particles. On the other hand, the photograph of the F2 formulation demonstrated smooth surfaced dense particles with improved solubility, which enabled a faster dissolution of TC through SDs. The visual surface morphology products of F4 and F5 demonstrated slightly laminated, film-like, fluffy, and porous particles that were smooth with an increased surface area of the solid-state particles. This most likely will increase the probability that the drug might encounter the hydrophilic carriers. Overall, the microphotographs demonstrated that the wetting effect of the drug along with the carriers formed micropore capillaries that facilitated the solubility of the drug and resulted in a faster dissolution of TC-dispersed SDs.

Table 4 shows no significant changes in drug content results after the completion of storage period. There was no significant variation in the drug concentration (p>0.05). Hence, the study confirms the formulations were stable at given condition.

**Figure 8:**
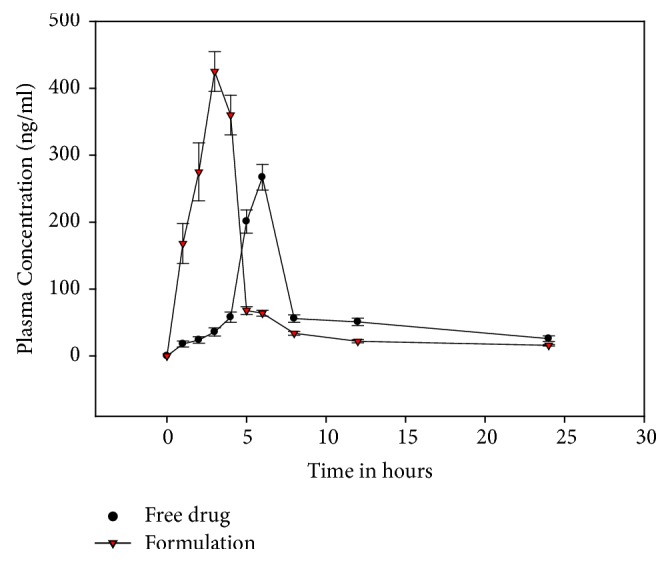
Plasma concentration time profile for TC and solid dispersion (1:2:6, TC: PEG-6000: *β*-CD) after oral administration to Sprague-Dawley rats.

We administered TC to the rats orally and measured the pharmacokinetic properties in vivo. In Figure 8, we plot the blood concentration-time profiles of TC administered orally at a dose of 10 mg/Kg body weight. Furthermore, we measured various pharmacokinetic parameters including C_max_, T_max_, and AUC 0–24 as listed in Table 5. Administered orally, the optimized SD of TC resulted in a gradual elevation of blood TC concentration up to C_max_ 453 ng/ml reached in 2.98 hours. In contrast, the free TC demonstrated a 2.3-hour delay in the drug absorption and C_max_ of 201 ng/ml that was achieved 5.0 hours after administration (Figure 7). Our findings agree with a previously reported T_max_ of 4 to 7 hours for free TC [27]. Past work has shown that the elimination of TC is biphasic with a first-phase half-life of 7 hours and a terminal half-life of 7-11 days [28–30]. As a result, TC has two values of elimination rate constant, K at 0.098 h-1 487 (initial half-life) and 0.004 h-1 381 (terminal half-life). The faster absorption, higher C_max_, and shorter T_max_ for SD might be attributed to higher in vitro solubility and dissolution efficiency which were reflected in the solubility and absorption of the drug.

The statistical analysis of our data demonstrated a significant difference between SD and the free drug with regard to the values of C_max_ (p < .05), T_max_ (p < .05), and AUC 0–24 (p < .05). We found a 1.4-fold increase in C_max_ and AUC of TC using the ternary solid dispersion technology. The relative bioavailability of ternary SD to the free drug was calculated to be ~106 % based on the AUC value of the orally administered TC. Our findings are consistent with the results of the dissolution study; the binary complex formulations showed the faster release of TC in comparison to single-polymer compositions and pure drug. However, further studies are needed to determine the aggregate effect of the drug tamoxifen and its metabolites in the blood circulation.

## 4. Conclusion

Solvent evaporation is one of the most effective techniques currently used to prepare solid dispersions containing TC. The method has a dramatic effect on improving the solubility of TC using hydrophilic carriers. Hydrophilic carriers of individual and combination of formulations in the phase solubility studies of TC demonstrated a good linear relationship of TC. We also noted an improved wetting effect that influences the formation of solid drug solutions most notably in *β*-CD formulations that showed a higher solubility profile as compared to PEG-6000. Our results showed that the products of F2, F4, and F5 displayed the best formulations based on their satisfactory results of solubility and dissolution. Furthermore, the obtained results from the DSC, FTIR, XRD, and SEM explained the disappearance of the crystallinity in the solid dispersions of these formulations. There was no significant variation in the drug concentration during different time intervals and the formulations were found to be stable. Overall, our work demonstrates enhanced dissolution and pharmacokinetic behavior in TC-loaded SD in PEG-6000 and *β*-CD formulations, both of which hold therapeutic promise. We believe that this improved and promising formula holds the potential to improve the pharmacological application and use of TC in cancer treatments. Additional studies are needed to evaluate the aggregate effect of the drug tamoxifen and its metabolites in blood circulation.

## Figures and Tables

**Table 1:** The phase solubility of TC PMs complexes in water and phosphate-buffered saline (PBS, pH 6.8, 7.4) at 37°C.

**Samples**	**Ratio**	**Solubility mg/ml**		
		**Water**	**6.8 pH PBS**	**7.4 pH PBS**
Drug	-	0.479±0.02	0.392±0.02	0.319±0.02
TC+PEG 6000 (F1)	1:2	0.834±0.02	0.719±0.03	0.862±0.01
TC+*β*-CD (F2)	1:2	0.846±0.04	0.913±0.01	0.915±0.03
TC+PEG6000+*β*-CD (F3)	1:2:2	0.853±0.03	0.946±0.05	0.834±0.01
TC+PEG6000+*β*-CD (F4)	1:2:4	0.888±0.03	0.989±0.05	0.889±0.01
TC+PEG6000+*β*-CD (F5)	1:2:6	0.987±0.04	1.324±0.05	1.156±0.03

**Table 2:** Composition of TC solid dispersions, product yield, and drug content.

**Formulations **	**TC**	**PEG-6000**	**β** **-CD**	%** yield **	%** Drug content **
F1	1	2	-	97.40±1.20%	98.43±1.79%
F2	1	-	2	99.20±0.42%	98.06±0.58%
F3	1	2	2	97.80±0.62%	98.64±1.12%
F4	1	2	4	98.52±1.40%	99.06±1.10%
F5	1	2	6	98.65±0.98%	98.81±1.12%

**Table 3:** The dissolution efficiency in percentage of TC binary complexes SDs.

**Formulations**	**DE% (±SD)**								
	Water			PBS pH 6.8			PBS pH 7.4		
**Minutes**→	10	20	30	10	20	30	10	20	30

F4	64±0.4	72±0.9	78±1.4	67±0.9	75±0.5	82±1.6	85±2.4	88±0.9	92±2.3
F5	82±0.9	86±0.5	88±1.3	85±0.9	88±0.3	92±0.8	92±3.4	94±4.1	96±2.5
TC	25±1.2	35±1.3	45±0.9	20±1.5	30±1.7	40±1.6	16±2.4	32±1.9	42±2.1

**Table 4:** Stability studies according to the ICH guidelines.

**Formulation**	**0 months**	**3 months**	**6 months**
**F2**	98.06±0.58%	97.66±0.68%	97.02±0.49%
**F4**	99.06±1.10%	98.28±0.94%	98.01±0.91%
**F5**	98.81±1.12%	97.91±1.01%	96.35±0.89%

**Table 5:** TC pharmacokinetics parameters in rats after oral administration of 10 mg/kg dose.

PK Parameter	TC SD	Free TC
C_max_ (ng/ml)	453 ± 42.02	299.4 ± 25.14
T_max_ (h)	2.98 ± 0.412	5.241 ± 0.32
AUC 0–24 (ng.h/ml)	2008.7 ± 98.25	1604.74 ± 124.09

C_max_: maximum plasma concentration; T_max_: time of maximum concentration; AUC 0–24: area under the curve of plasma concentration versus time from t = 0 to 24 h after administration. Values are expressed as mean ± SD (n=6).

## Data Availability

The data used to support the findings of this study are included within the article such as FTIR, XRD, DSC, in vitro release studies, and so forth.
